
JOURNAL INFORMATION
==============================
NLM Title Abbreviation: ScientificWorldJournal
Journal ID: ScientificWorldJournal
NLM Journal ID: 101131163
Journal ID: TSWJ

The Scientific World Journal

ISSN: 2356-6140
EISSN: 1537-744X
Publisher: Wiley

ARTICLE INFORMATION
==============================
PMCID: PMC6009371
PMID: 29973784
DOI: 10.1100/tsw.2002.10
Article ID: 546029
Article version: 1
Subjects: Short Report

Candidate Gene in Predicting In Vivo Ovarian Cancer Response to Combination Therapy with Paraplatin and Paclitaxel

Mirhashemi Ramin 1 *
Arena J. Fernando 1
Frudakis Tony 2
Lambrou Nicholas 1
Arboleda Jane 1
Hunt Marsha 1
Medranda Maria 1
Averette Hervy 1
Penalver Manuel 1
Department of Obstetric & Gynecology, University of Miami School of Medicine, P.O. Box 016960, Miami, FL 33136, USA
DNAPrint Genomics, Inc., 900 Cocoanut Ave., Sarasota, FL 34236, USA
*Ramin Mirhashemi: rmirhashemi@med.miami.edu
Publication date: 2002
Electronic publication date: 2002 Jun 15
Volume: 2
First page: 19
Last page: 20
Copyright: Copyright © 2002 Ramin Mirhashemi et al.
Copyright year: 2002
License: This is an open access article distributed under the Creative Commons Attribution License, which permits unrestricted use, distribution, and reproduction in any medium, provided the original work is properly cited.
License URL: https://creativecommons.org/licenses/by/3.0/

==============================
Advances in Gene Technology: The Genome and Beyond –
Structural Biology for Medicine (Proceedings of the 2002 Miami
*Nature Biotechnology* Winter Symposium)
*TheScientificWorld* 2002, 2(S2), 19–20
ISSN 1532-2246; DOI 10.1100/tsw.2002.10

### CANDIDATE GENE IN PREDICTING *IN VIVO* OVARIAN CANCER RESPONSE TO COMBINATION THERAPY WITH PARAPLATIN AND PACLITAXEL

Ramin Mirhashemi[1], J. Fernando Arena[1], Tony Frudakis[2], Nicholas Lambrou[1], Jane Arboleda[1], Marsha Hunt[1], Maria Medranda[1], Hervy Averette[1], and Manuel Penalver[1]
[1]Department of Obstetric & Gynecology, University of Miami School of Medicine, P.O. Box 016960, Miami, FL 33136; [2]DNAPrint Genomics, Inc., 900 Cocoanut Ave., Sarasota, FL 34236
*rmirhashemi@med.miami.edu

**INTRODUCTION**. Adverse drug reactions, due at least in part to interindividual variability in drug response, rank between the 4th and 6th leading causes of death in the U.S. The field of “pharmacogenetics” (the study of variability in drug response due to heredity), should help in reducing drug-caused morbidity and mortality[1]. The underlying genetics for the efficacy of the chemotherapeutic treatment of ovarian cancer is still unknown and subject to a high degree of uncertainty and interindividual variability[2]. In the present project, using a pharmacogenomic approach, we evaluate the patient response rates to primary chemotherapeutic treatment of ovarian cancer with combination of paclitaxel and the carboplatin agents. In an attempt to define the genetic determinants for this variability, we are conducting a pharmacogenomics study to predict response based on patient genomics[3].

Our results demonstrate that single nucleotide polymorphic (SNPs) in candidate genes being identified could potentially be used pre-treatment to individualize chemotherapy for patients with ovarian cancer.

**METHOD.** Our approach is a systematic haplotype-based candidate gene approach, where we genotype each patient at each SNP site for each gene known to be relevant for xenobiotic disposition, then computationally infer haplotypes, and geometrically model the data in order to identify associations between haplotypes and response. We have chosen this novel approach to objectively define an optimal pharmacogenomics solution.

**RESULTS.** We have constructed SNP maps for 50 genes known to be involved in xenobiotic metabolism and/or the disposition of paclitaxel and carboplatin and have discovered an average of 30 candidate SNPs per gene, which, upon polymorphism screening, validated at a rate of about 18 SNPs per gene. Having obtained genotypes for 57 patients at each of the SNPs in several genes, preliminary results have revealed a statistically significant association (Fishers Exact $p = 0.00332 \pm 0.00043$) between haplotypes at the CYP3A4 gene and a lack of response to paclitaxel/carboplatin as measured by the CA-125 tumor marker. In particular, the CGC and CGT CYP3A4 haplotypes were only present in the nonresponder group (n = 22 individuals, 106 readings), when a 50% decrease in CA-125 was used to define responders (n = 9 individuals, 37 readings). When a 20% decrease in CA-125 was used, a similar result was obtained (Fishers Exact

*p* = 0.016 ± 0.001); CGC was found in the nonresponder group 75% of the time, and the CGT haplotype was found in the nonresponder group 100% of the time.

**DISCUSSION.** In the present study, we have investigated SNPs in 50 candidate genes involved in the xenobiotic metabolism/disposition of paclitaxel and carboplatin[4]. We identified and validated SNPs in a reference population, and screened them for the ability to resolve between paclitaxel and carboplatin responders and nonresponders. We have observed 50% reductions in CA-125 tumor marker values are associated with two haplotypes (CGC and CGT) in the CYP3A4 gene. Even when a 20% decrease in CA-125 was used as the criteria for response grouping, rather than a 50% decrease, the two haplotypes were associated with nonresponse. The same results were obtained whether average response per patient was used or whether individual readings were grouped. Patient accrual is still in process and no similar data was found in the literature for comparison. This *in vivo* assay could represent a component of a classifier that could help classify ovarian cancer patients as potential responders or nonresponders to our conventional chemotherapeutic agents.

### REFERENCES

1. MEDLINE ABSTRACTS - CLINICAL IMPACT OF PHARMACOGENOMICS. What's the latest information on the clinical impact of pharmacogenomics? http://molecularmedicine.medscape.com/26893.rhtml
2. Goddard, K., Hopkins, P., Hall, J., and Witte, J. (2000) Am. J. Hum. Genet. 66, 216–234.
3. Shou, M., Lu, T., Drausz, K., Sai, Y., Yang, T., Korzekwa, K.R., Gonzalez, F., Gelboin, H. (2000) Eur. J. Pharmacol. 394(2–3), 199–209.
4. Dai, D., Zeldin, D.C., Blaisdell, J., Chanas, B., Coulter, S., Ghanayem, B., and Goldstein, J. (2001) Pharmacogenetics 11(7), 597–607.
